# Electrospun Poly(Styrene−Co−Vinylbenzyl Chloride−Co−Acrylonitrile) Nanofiber Mat as an Anion Exchange Membrane for Fuel Cell Applications

**DOI:** 10.3390/polym14163236

**Published:** 2022-08-09

**Authors:** Dongho Kang, Ji Su Lee, Hyon Hee Yoon, Chinta Mani Sharma, Gautam Das, Young Soo Yoon

**Affiliations:** 1 Department of Chemical and Biological Engineering, Gachon University, Seongnam 461-701, Korea; 2 Department of Chemistry, Biswanath College, Biwanath Charialli 784176, India; 3 Department of Polymer Science and Engineering, School of Applied Science, Kyungpook National University, Daegu 41566, Korea; 4 Department of Materials Science and Engineering, Gachon University, Seongnam 461-701, Korea

**Keywords:** anion exchange membrane, styrene-co-vinylbenzyl, chloride-co-acrylonitrile, electrospinning, nanofiber mat, ion conductivity

## Abstract

A nanofiber mat of styrene−co−vinylbenzyl chloride−co−acrylonitrile copolymer as an anion exchange membrane (AEM) was synthesized via the electrospinning of organic reaction mixtures. The synthesized membranes were characterized using FT-IR spectroscopy for structural analysis. The AEM demonstrated a high ionic conductivity mainly due to the phase segregation in the membrane structure, as analyzed by transmission electron microscopy (TEM). The membrane properties such as water uptake, swelling ratio, and ion exchange capacity, as well as ionic conductivity, varied with the chemical composition. With the molar ratio of styrene, vinylbenzyl chloride, and acrylonitrile at 3:5:2, the highest ionic conductivity of 0.214 S cm^−1^ at 80 °C was observed. Additionally, the AEM retained 94% of original conductivity after 72 h of soaking in 1 M KOH solution.

## 1. Introduction

Fuel cells, which convert chemical energy directly to electrical energy, have gathered noticeable attention for their durability, high energy density and energy conversion efficiency, and benign byproducts [1]. One of the most widely investigated low-temperature fuel cells is the proton exchange membrane fuel cell (PEMFC), subjected to commercialization for stationary and mobile applications. The PEMFC was adopted as a power source for the Apollo space program and showed potential as a stationary energy producer [2]. This has led to the alternation of conventional combustion engines for personal automobiles and public buses into fuel cell-powered electric motors, which are vividly developing in global society [3]. The PEMFCs, however, suffer from drawbacks, such as the extensive usage of expensive noble platinum catalysts and Nafion membrane, fuel crossover, and the widespread corrosion of cell compartments due to the highly acidic environment [4,5,6]. In contrast, the anion exchange membrane fuel cell (AEMFC) exhibits the potential to substitute the PEMFC, owing to the use of inexpensive catalysts, fast reaction kinetics [7], low or negligible fuel cross-over, and less of an corrosive environment [7,8]. Furthermore, AEMFC exhibits fuel flexibility; for instance, various kinds of fuel, such as methanol [9,10], borohydride [11,12], and urea [13,14] have been applied in fuel cells due to their high energy density, abundance in nature, and safer handling.

However, compared to Nafion, a significant improvement in the ionic conductivity of the AEM is essential [15,16]. However, owing to the lower mobility of hydroxide ions (OH^-^) compared to hydrogen ions and the low dissociation constant of a quaternary group compared with the sulfonic-acid group, AEMs generally show poor ionic conductivity [7]. To circumvent the low mobility of the OH^-^, a high ion exchange capacity (IEC) in the membrane matrix has been effectively applied to enhance the ionic conductivity. Although a high IEC can provide better ionic conductivity, the membrane is prone to degradation, and disintegration occurs at increased temperatures. This type of issue can seriously affect the fuel cell operation. So, balancing the ionic conductivity and, at the same time, maintaining the mechanical and chemical stability of the membrane in the hydrated state at operational temperatures has become one of the foremost challenges in the field of AEMFC. Thus, several different types of AEMs with remarkable enhanced properties (high ionic conductivity, good mechanical properties, and superior chemical stability) have been reported, such as a copolymer of vinylbenzyl chloride, fluoropoly(olefin), radiation-grafted polyethylene, and ethylene tetra fluoro ethylene (ETFE);phase-separated morphology obtained with a side-chain polymer backbone; and polymer nano-composites [15]. Aside from the IEC, phase segregation in the micro or nanoscale in the AEM structure has been observed to greatly enhance the ionic conductivity in AEMs [14,15]. This has led to the development of AEMs with a structure like Nafion.

Moreover, AEMs contain quaternary ammonium groups induced by bromination or chloromethylation followed by quaternization [14,15]. The common chloromethylation reaction for the synthesis of AEMs uses chloromethyl ethyl ether, which is highly carcinogenic [17,18]. To avoid this situation, vinylbenzyl chloride, which possesses a chlorine functional group, can be utilized to skip the dangerous chloromethylation reaction step [15]. The benzene ring with the ether-free structure of styrene increases the heat and chemical stability at high pH conditions, whereas the presence of acrylonitrile in the membrane structure imparts the flexibility of the membrane [19,20]. Julia et al. demonstrated an AEM with good alkali stability by using the vinylbenzyl chloride monomer with radiation grafting synthesis [21]. Bo et al. showed a cross-linked styrene-acrylonitrile-bis-imidazolium functionalized ionic liquid monomer AEM that achieved a hydroxide conductivity of 2.0× 10^−^^2^ S cm^−^^1^ with great conductivity retainment after 30 days immersion in 1 M KOH solution [22].

Electrospinning is a cost-effective technique that has recently attracted attention for the fabrication of AEMs [23]. A fibrous mat-like structure can be obtained by electrospinning, which ejects the fiber from the polymer solution on the application of a high voltage at the tip of the connected syringe [23,24]. These nanofibers with a high surface area form a porous network providing a high charge density and the ease of the diffusion of the mobile species. Fabrications of the AEM with the electrospinning technique have not been explored widely [17,25,26]. Thus, it is expected that an AEM fabricated by electrospinning not only results in the formation of a large surface area inducing high ion transfer rates but can be applied at an industrial scale for the fabrication of a well-controlled structure and size. The nanofibrous AEM mat has shown promising performance in inducing a good performance enhancement in case of PEMs; however, there are plenty of opportunities to develop AEM with different chemical compositions, and the study electrostatic field effect on the fiber is still not fully understood. The water uptake and ion exchange capacity (IEC), which are important parameters of the ionic conducting membrane, can be increased due to the spacious volume created by the stacking structure of nanofibers within the membrane [14].

In this work, a novel AEM with a fibrous mat-like structure was synthesized via the electrospinning technique using a co-polymerized solution with different compositions of styrene, vinylbenzyl chloride, and acrylonitrile. A series of membranes showed potential as an alkali-stable, high ionic-conducting characteristic. The membrane properties including water uptake, swelling ratio, ionic conductivity, and stability were investigated explicitly. The FT−IR spectra of a series of the membrane are demonstrated. The stacked fibrous structure of membranes was observed by the SEM and TEM.

## 2. Materials and Methods

### 2.1. Materials

Styrene, vinylbenzyl chloride, acrylonitrile, N,N-dimethylformamide (DMF), sodium tungstate, benzoyl peroxide (BPO), and trimethylamine (TMA) were purchased from Sigma Aldrich (Yongin-si, Korea). All the chemicals used in the present investigation are of reagent grade.

### 2.2. Synthesis of Styrene−Co−Vinylbenzyl Chloride−Co−Acrylonitrile

**Scheme 1:**
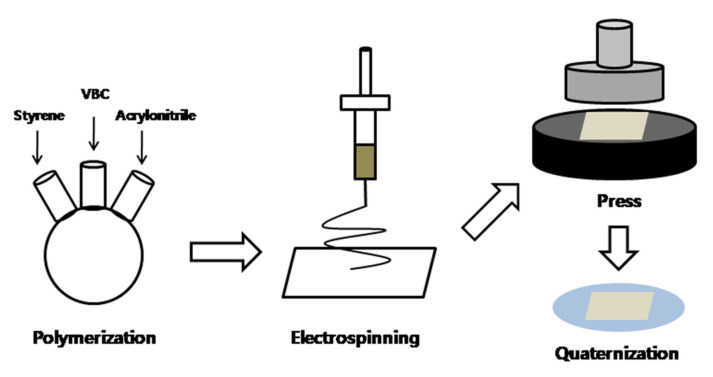
Experimental process for the fabrication of the series of membrane from polymerization to quaternization.

The experimental process is schematically depicted in Scheme 1. Styrene, vinylbenzyl chloride, and acrylonitrile monomers were mixed at a desired molar ratio in DMF solvent in a three-neck-flask. Then, BPO (0.5 wt% of the total weight of monomers) was added as a polymerization initiator and stirred for 24 h at 70 °C under inert atmosphere. The resulting yellowish viscous solution was then poured into the excess of methyl alcohol, and the precipitate was collected after filtration. The filtrate was washed repeatedly with methyl alcohol and dried in a vacuum oven for 24 h at 45 °C. The products with different compositions of styrene (S), vinylbenzyl chloride (V), and acrylonitrile (A) (i.e., S/V/A = 5/3/2, 4/4/2, and 3/5/2) were prepared and marked as SVA532, SVA442, and SVA352.

### 2.3. Fabrication of AEM by Electrospinning

The synthesized S−V−A co-polymer (1.6 g) was dissolved in DMF solvent (2 mL) to achieve a suitable viscosity for electrospinning. The well-dissolved polymer solution was poured into the special syringe of an electrospinning setup at 16.0 kV, with a 15 cm distance between the tip and the collector (Scheme 1). The cylindrical collector was wrapped in aluminum foil and rotated at a speed of 150 rpm. The rotating axis of the collector moved back and forth repeatedly with a speed of 2 mm s^−1^. The polymer nanofibers were formed by electrospinning at a pumping rate of 2 μL s^−1^ and were collected on a cylindrical stainless drum covered by an aluminum foil rotating at 150 rpm. The rotating axis was moved back and forth repeatedly at a speed of 2 mm s^−1^ by 10 mm from the center of the collector. The electrospun mat was peeled off from the aluminum foil and pressed at approximately 1500 psi for 30 s (Scheme 1).

### 2.4. Quaternization

The pressed S−V−A co-polymer membrane was quaternized with the TMA solution. Briefly, the pressed membrane was immersed into a TMA solution (at five times of the mole of V in the S−V−A membrane sample) for 24 h at 40 °C under continuous stirring. The membrane was then washed with deionized (DI) water several times to remove the unreacted TMA. Afterwards, the membrane was converted to hydroxide form under nitrogen-purging by immersion in 1 M KOH aqueous solution. The quaternized S−V−A co-polymer membrane was denoted as qSVA. The AEM samples were stored in a vacuum desiccator before use.

### 2.5. Analysis

A Fourier transform infrared spectrometer (FT−IR, Vertex 70/Raman, Bruker, Billerica, MA, USA) was used to characterize the functional groups in the polymer membrane samples. The morphological structure of membranes was investigated using a scanning electron microscope (SEM, S-4700, Hitachi S-4700, Tokyo, Japan) and a transmission electron microscope (TEM, Tecnai G2 F30 S-Twin, AP Tech, Incheon Incheon, Korea). The membrane samples were immersed in 1 M Na_2_WO_4_ solution for 24 h at 30 °C and dried in a vacuum oven after washing with DI water to prepare TEM samples. A thermogravimetric analyzer (TGA, TA Instruments SDT Q600, New Castle, DE, USA) was used to examine the tensile strength and thermal stability of the membranes. The TGA temperature was raised at 10 °C min^−1^ until 600 °C with nitrogen flowing.

### 2.6. Characterization

A Fourier transform infrared spectrometer (FT−IR; JASCO FT-IR 300E, Tokyo, Japan) was used to record the FT−IR spectra of the samples. The morphological characterization was carried out by a scanning electron microscope (SEM, Hitachi S-4700, Tokyo, Japan) and a transmission electron microscope (TEM, TecnaiG2 F30 S-Twin, AP Tech).The TEM samples were prepared by ultramicrotome (UMT, PT PC Ultramicrotome&Photographic, RMC, York City, NY, USA) immersed into 1 mol L^−1^ Na_2_WO_4_ solution at 30 °C for 48 h. Then samples were washed with HPLC water several times and dried in a vacuum oven at 30 °C for 24 h. The thermogravimetric analyzer (TGA, TA Instruments SDT Q600, New Castle, DE, USA) was used to investigate the thermal stability of the membranes.

### 2.7. Water-Uptake (WU),Swelling Ratio (SR), and Ion Exchange Capacity (IEC)

The water uptake (*WU*) and swelling ratio of in-plane (*SR_ip_*) and through-plane (*SR_tp_*) were measured by comparing the change in the weight and dimension of the membrane sample difference before and after soaking into the water [14]. The soaked samples were dried in the vacuum oven at 50 °C before being measured. The *WU*, *SR_ip_*, and *SR_tp_* were calculated according to the following equations.

(1) $$WU\left(\%\right)=\frac{W_{w}-W_{d}}{W_{d}}$$

(2) $$SR_{ip}\left(\%\right)=\frac{L_{w}-L_{d}}{L_{d}}$$

(3) $$SR_{tp}\left(\%\right)=\frac{T_{w}-T_{d}}{T_{d}}$$

where $W_{w}$ is the weight of the wet membrane, $W_{d}$ is the weight of the dried membrane, $L_{w}$ is the width of the wet membrane, $L_{d}$ is the width of the dried membrane, *T_w_* is the thickness of the wet membrane, and *T_d_* is the thickness of the dried membrane.

The ion exchange capacity (*IEC*) was measured by an acid-base titration method [14]. Samples cut into specific sizes were immersed into a 0.01 M NaOH solution for 24 h at room temperature and washed with DI water. The washed membranes were then equilibrated with an aliquot of 0.01 M HCl solution. The amount of OH^−^ ions liberated was estimated by acid–base titration.The *IEC* was calculated based on the titrated volume, the mole concentration, and the weight before and after the drying.

(4) $$IEC\left({\mathrm{mmolg}}^{-1}\right)=\frac{C_{NaOH}\times \left(V_{B}-V_{S}\right)}{W_{d}}$$

where $C_{NaOH}$ is the mole concentration of the NaOH, $V_{B}$ is the volume of the blank solution, $V_{S}$ is the volume of the sample’s solution, and $W_{d}$ is the weight of the dried sample.

### 2.8. Ionic Conductivity (σ)

The AEM sample was cut into 2.5 cm × 2.5 cm. The specially manufactured zig with a platinum conductor surface area of 1 cm^2^ held the anion exchange membrane during the electrochemical impedance spectroscopy (EIS) measurements. From room temperature, the ionic conductivities were measured until 80 °C under fully hydrated and nitrogen purging to prevent carbon dioxide contamination. The ionic conductivity was calculated with the equation below [14]:

(5) $$\sigma \;=\frac{D}{R\times A}$$

where R (Ω) is the resistance, A (cm^2^) is the surface area of the sample, and D (cm) is the distance between the electrodes.

### 2.9. Alkaline Stability

A series of AEM samples (2 cm × 2 cm) were soaked into 1 M KOH solution for 9 days. After 3 days, one AEM sample was taken at 216 h, and ionic conductivity was measured every 72 h. The KOH solution was replaced witha newly mixed solution after an ionic conductivity test every 72 h to maintain the constant concentration of the alkaline ion.

## 3. Results and Discussion

### 3.1. Characterization: Structure and Morphology

**Figure 1:**
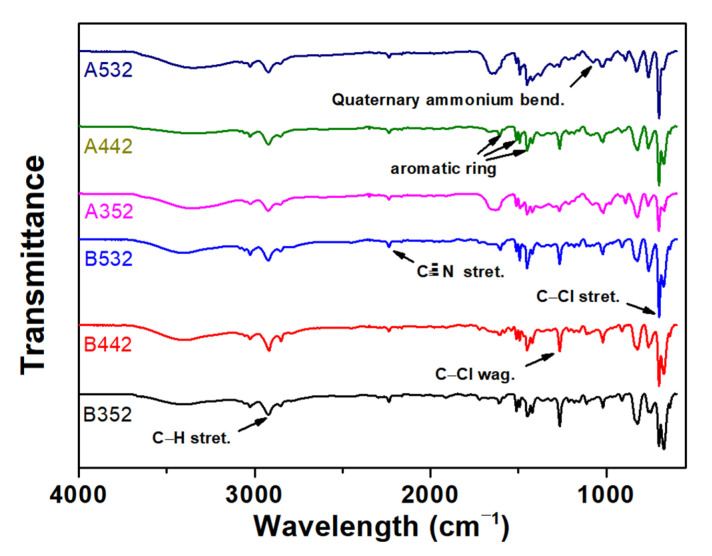
FT−IR spectrum of S−V−A copolymer membranes before (SVA352, SVA442, SVA532) and after the quaternization (qSVA352, qSVA442, qSVA532) of membranes.

Figure 1 shows the FT−IR spectrum of electrospun S−V−A copolymers before and after quaternization. The C−H stretching was observed in the spectra at 3000−2760 cm^−1^ for all the samples, which was due to the main polymer chain. The presence of vinylbenzyl chloride and acrylonitrile in the polymer structure was identified by the observance of C−Cl wagging (1265 cm^−1^), C−Cl stretching (700 cm^−1^), and C−N triple bond stretching (2220 cm^−1^) in the spectra [27]. Aromatic group peaks at 1610, 1511, 1446 cm^−1^, 1490 cm^−1^, and 1080 cm^−1^ [28,29] evolved after quaternization with TMA. Further, the diminishing intensities of C−Cl wagging and C−Cl stretching bands in the quaternized samples were indicative of the successful attachment of the amine group to the polymer backbone by the substitution reaction.

**Figure 2:**
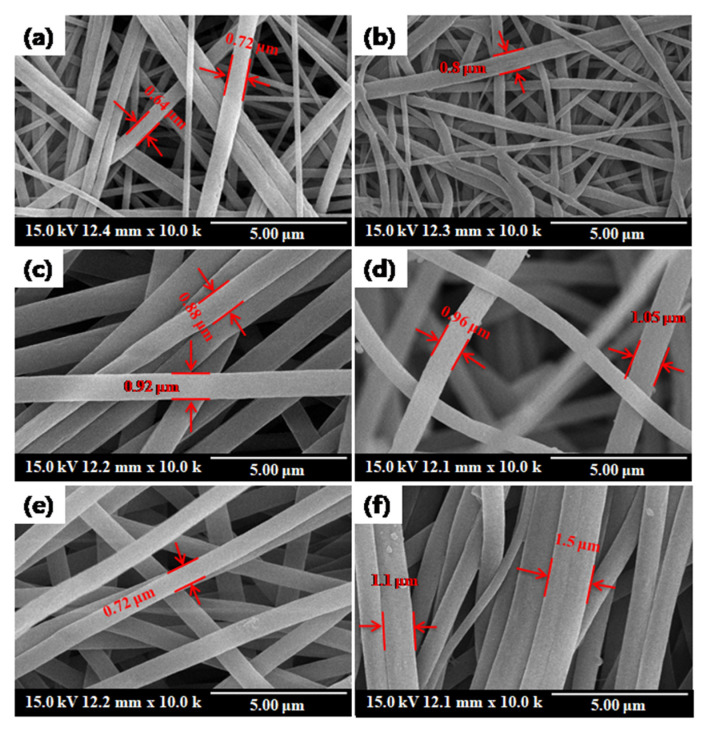
SEM images of electrospun membranes: SVA352 (**a**), qSVA352 (**b**), SVA442 (**c**), qSVA442 (**d**), SVA532 (**e**), and qSVA532 (**f**).

The morphology of the electrospun membrane possesses a fiber-mat structure with a high surface area consisting of sub-micron-sized fibers, as shown in the SEM images (Figure 2). The membranes consisted of a stacked fibrous structure from the top to the bottom, and the porous structure was distributed throughout the matrix, which might lead to the expansion of ionic channels. The diameter of the polymer fibers was in thesub-micron range. The fiber diameter increased after quaternization for all three AEM samples with different S−V−A compositions, mainly because quaternization improved the hydrophilicity of the S−V−A co-polymer, which facilitates water absorption, resulting in the swelling of the fibers [15].

**Figure 3:**
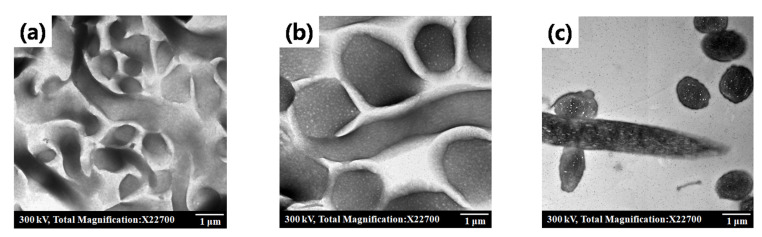
TEM images of the quaternized electrospun membrane: (**a**) qSVA352, (**b**) qSVA442, and(**c**) qSVA532.

The structural morphology of the AEM samples was further analyzed using TEM. The TEM samples were pretreated with the sodium tungstate solution to stain the hydrophilic domains which appear dark in the TEM images; thus, the hydrophilic and hydrophobic phases in the sample can be distinguished from the TEM images. The phase segregation is beneficial for the transport of hydroxyl ions inside the membrane [14]. As shown in the TEM images (Figure 3), dark and bright regions were observed to clearly correspond to the hydrophilic and hydrophobic domains, respectively, which were ascribed to the ionic groups and polymer backbone, respectively. This distinction indicates phase segregation between the hydrophilic and hydrophobic phases, forming ionic channels in the membrane [30,31,32]. As seen in the TEM image, among the AEM samples, qSVA352 exhibited the most well-distributed and connected phase-segregated morphology, implying that qSVA352 possessed more well-connected ionic channels than the other two membranes, which facilitated a higher ion mobility, translating into a higher ionic conductivity [33,34,35].

### 3.2. Membrane Properties: WU, SR, and IEC

*WU* has a substantial influence on the membrane properties, such as swelling and ionic conductivity. A high *WU* induces a high swelling, which reduces the membrane dimensional stability and destabilizes the coating of the catalysts, but the water in the membrane is important for ion transport. Thus, it is pertinent to judiciously control the water content of the membrane. The *WU* of the membrane in this work was evaluated with respect to the varying concentration of the vinylbenzyl chloride in the copolymer composition. As presented in Table 1, the *WU* of the membrane increased significantly as the content of vinylbenzyl chloride in the copolymer increased, mainly because the vinylbenzyl chloride, which is quaternized, increases the hydrophilicity of the membranes. However, no large swelling occurs due to the innate structural advantage of the electrospun material. Further, the porous structure helped the membrane to hold the water in between fibers.

The *WU* and *SR* are influenced by the charge density in the membrane structure; thus, the *IEC*s of the membranes were determined. As shown in Table 1, the *IEC*s of the membrane samples were 0.786−1.862 mmol g^−1^ depending on the monomer composition. The AEM sample with higher vinylbenzyl chloride content exhibited a higher *IEC* value, most probably because the vinylbenzyl chloride provides sites for the substitution to quaternary ammonium.

### 3.3. Ionic Conductivity

Ionic conductivity (σ) is considered to be the most important property of AEMs since achieving a high σ is necessary to achieve a high power density in fuel cells [34]. It was reported that σ can be enhanced by inducing a higher *IEC* and a good phase separation in the membrane structure [33]. As expected, qSVA352, with the highest *IEC* and *WU*, exhibited the highest σ compared to the other membranes at all temperatures. The σ of qSVA352 at room temperature was 0.132 S cm^−1^, which is 4.3−fold higher than the σ of qSVA532 (0.031 S cm^−1^). Additionally, qSVA352 exhibited better phase separation in the membrane structure, as observed from the TEM image, indicating that σ was also strongly affected by the phase separation structure due to the formation of well-connected ionic channels inside the SVA352 membranes. The highest σ of 0.214 S cm^−1^ was achieved with the qSVA352 electrospun membrane at 80 °C. These σ values are higher than the previously reported values [13,14,16].

**Figure 4:**
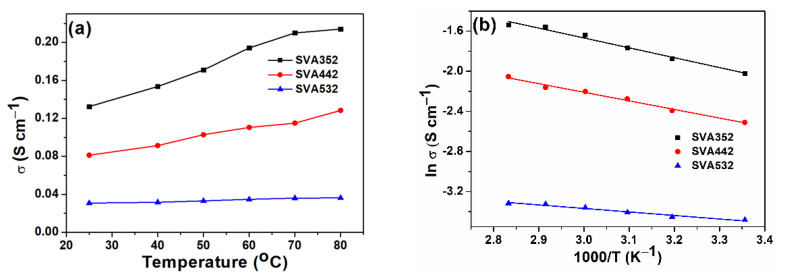
(**a**) Ionic conductivity vs temperature and (**b**) Arrhenius plot of qSVA352, qSVA442, and qSVA532.

Figure 4 shows the Arrhenius plot of the σ values of different AEM samples. From the Arrhenius plot, the activation energies (*E*_a_) of qSVA352, qSVA442, and qSVA532 were calculated to be 8.17, 7.12, and 2.92 kJ mol^−1^, respectively. The low *Ea* of qSVA532, as compared to other AEMs in this work, indicated the higher mobility of the hydroxide ions at the interfaces, which is due to a greater number of charged species owing to the higher concentration of vinylbenzyl chloride in the copolymer composition [14,36].

### 3.4. Thermal and Chemical Stability

**Figure 5:**
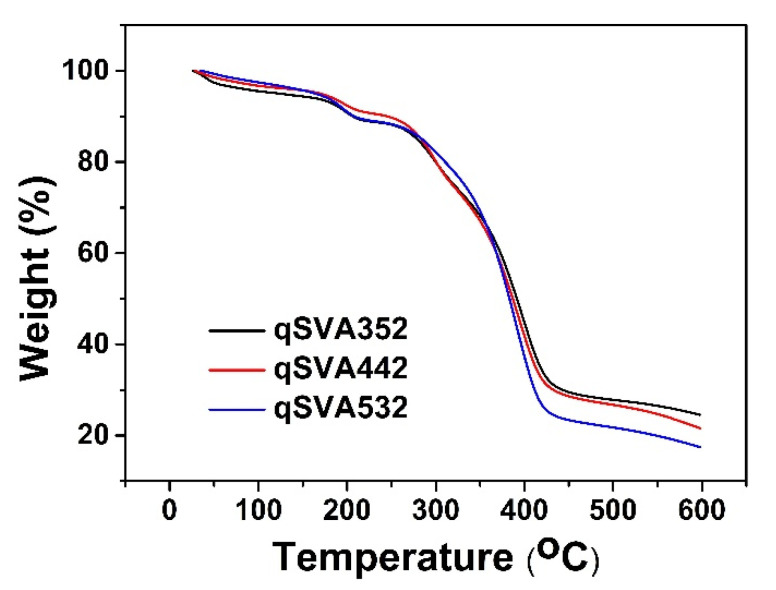
Thermogravimetric analysis of qSVA352, qSVA442, and qSVA532.

The thermal stability of the AEM sample was measured in the range of 25 °C to 600 °C (Figure 5). The water was completely evaporated during the first weight loss at the beginning and the second weight drop at 175 °C. After that, the functional groups of the membrane started to degrade. The polymer backbone was then collapsed after 400 °C. The result suggested that these S−V−A copolymer membranes are thermally stable at ~200 °C. As shown in the TGA graph, the weight loss trends of all three membranes were similar, because they contained the same total number of styrene and vinylbenzyl chloride monomers. Both styrene and vinylbenzyl chloride contain a benzene group, which is considered to contribute to the thermal stability of the co-polymer [19].

**Figure 6:**
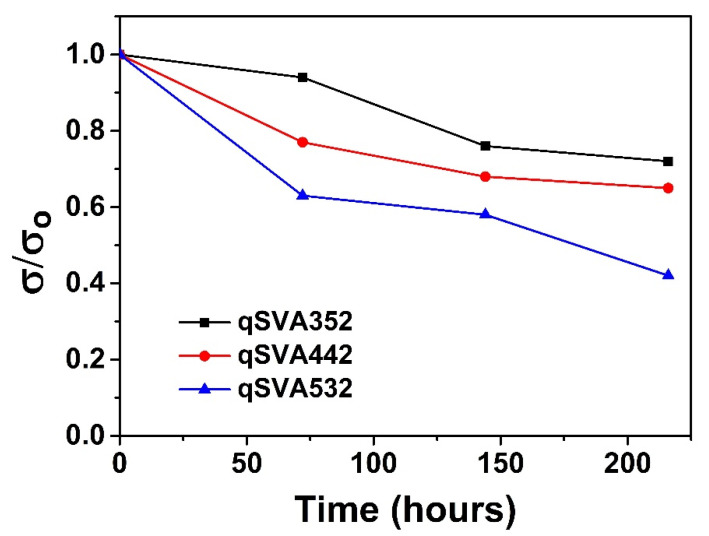
Alkaline stability of qSVA352, qSVA442, and qSVA532 in 1 M KOH solution for 200 h.

The membrane samples were soaked into the 1 M solution for over 200 h, and the ionic conductivity was measured every 72 h (Figure 6). The qSVA352 membrane retained 94% of the original ionic conductivity after 72 h; on the other hand, the qSVA532 membrane retained only 63% (Figure 6). The most probable reason for the higher ionic conductivity retention ofqSVA352 is that it exhibited better alkaline stability compared toqSVA532; this can be related to the stability of the TMA under alkaline conditions. The qSVA352 membrane was highly substituted with TMA due to its higher content of vinylbenzyl chloride than qSVA532. It is has been reported that the N^+^ with three or more carbon atoms is stable under alkaline exposure [37]. With the stable characteristic of TMA, a high mole percentage of the TMA in the SVA352 compared to the other two membranes could lead to better alkaline stability.

## 4. Conclusions

The nanofiber-mat type styrene-co-vinylbenzyl chloride-co-acrylonitrile was synthesized via the electrospinning of the copolymer solution. The FT-IR spectra demonstrated the composition of a series of the membrane. The stacked-fibrous structure and the spacious area within the membrane for the ion media were observed from the SEM. The enlarged diameter of the fiber after the quaternization indicates the water absorption with the increased hydrophilicity of the membrane. The TEM analysis showed dark and bright contrast regions, which indicated phase segregation for enhanced ion transportation, and qSVA352 showed the most distinct channels compared to the other membranes. The synthesized membrane featured a high *WU* without much expansion with its observed sufficient area that can store the media within the membrane. The highest ionic conductivity achieved in this work was 0.214 S cm^−1^ at 80 °C with the qSVA352 membrane. The membrane is stable at the temperature range of ~150 °C, which is approximate to the cell’s operating temperature. Furthermore, qSVA352 retained 94% of the original ionic conductivity even after being immersed in 1 M KOH for 72 h, and its high retention rate is greatly related to the stability of tangled trimethylamine under alkaline conditions. Thus, the electrospinning of the styrene-co-vinylbenzyl chloride-co-acrylonitrile copolymer solution can be utilized for the fabrication of anion exchange membranes with a high conductivity and stability.

## Figures and Tables

**Table 1:** Membrane properties at room temperature.

Sample	*WU* (%)	*SR_ip_* (%)	*SR_tp_* (%)	*IEC* (mmol g^−1^)
qSVA352	425	7.8	50	1.862
qSVA442	281	3.7	33	1.319
qSVA532	222	2.7	14	0.786

## Data Availability

The study did not report any data.
